# A U.S. NATIONAL PROFILE OF OLD ADULTS WITH COGNITIVE IMPAIRMENT ALONE, PHYSICAL FRAILTY ALONE, AND BOTH

**DOI:** 10.1093/geroni/igz038.1467

**Published:** 2019-11-08

**Authors:** Meiling Ge, Qianli Xue

**Affiliations:** 1 Center on Aging and Health, Johns Hopkins Medical Institutions, Baltimore, Maryland, United States; 2 Department of Medicine Division of Geriatric Medicine and Gerontology, School of Medicine, Johns Hopkins University, Baltimore, Maryland, United States

## Abstract

Using data from NHATS, we aimed to identify characteristics (demographics, health conditions/events, self-care behaviors, psychological wellbeing) that distinguish joint vs. separate presence of physical frailty (by the Fried’s) and cognitive impairment (CI: bottom quintile of test performance in executive function and memory; or proxy-report of dementia diagnosis or AD8 score >=2). Of the 7,497 older adults, 25.5%, 5.6%, and 8.7% had CI only, frailty only, and both, respectively. After adjusting for demographic characteristics, current smoker, single disease, and knee surgery history uniquely identified “frailty only”. Although none was found to uniquely identify “CI only” or “both”, surgery history and comorbidity were strongly associated with “frailty only” and, to a lesser degree, “both”, but not “CI only”. The findings advocate for treating physical frailty and CI as overlapping yet distinct conditions, and prioritizing comorbidity, surgery history, and smoking status in clinical screening of frailty and CI before formal diagnostic assessments.

